# A Metabolomic Study of Rats with Doxorubicin-Induced Cardiomyopathy and Shengmai Injection Treatment

**DOI:** 10.1371/journal.pone.0125209

**Published:** 2015-05-04

**Authors:** Yu Chen, Yong Tang, Ya-Chen Zhang, Xiao-Hong Huang, Yu-Quan Xie, Yin Xiang

**Affiliations:** The Division of Cardiology, Xin Hua Hospital, Affiliated to Shanghai Jiao Tong University School of Medicine, Shanghai, 200092, China; University-Hospital of Parma, ITALY

## Abstract

Doxorubicin-induced cardiomyopathy (DOX-CM) is a severe complication of doxorubicin (DOX) chemotherapy. Characterized by cumulative and irreversible myocardial damage, its pathogenesis has not been fully elucidated. Shengmai Injection (SMI), a Traditional Chinese Medicine, may alleviate myocardial injury and improve heart function in the setting of DOX-CM. As a result of its multi-component and multi-target nature and comprehensive regulation, the pharmacological mechanisms underlying SMI’s effects remain obscure. The emerging field of metabolomics provides a potential approach with which to explore the pathogenesis of DOX-CM and the benefits of SMI treatment. DOX-CM was induced in rats via intraperitoneal injections of DOX. Cardiac metabolic profiling was performed via gas chromatography/mass spectrometry and ultra-performance liquid chromatography/tandem mass spectrometry. A bioinformatics analysis was conducted via Ingenuity Pathway Analysis (IPA). Eight weeks following DOX treatment, significant cardiac remodeling, dysfunction and metabolic perturbations were observed in the rats with DOX-CM. The metabolic disturbances primarily involved lipids, amino acids, vitamins and energy metabolism, and may have been indicative of both an energy metabolism disorder and oxidative stress secondary to DOX chemotherapy. However, SMI improved cardiac structure and function, as well as the metabolism of the rats with DOX-CM. The metabolic alterations induced via SMI, including the promotion of glycogenolysis, glycolysis, amino acid utilization and antioxidation, suggested that SMI exerts cardioprotective effects by improving energy metabolism and attenuating oxidative stress. Moreover, the IPA revealed that important signaling molecules and enzymes interacted with the altered metabolites. These findings have provided us with new insights into the pathogenesis of DOX-CM and the effects of SMI, and suggest that the combination of metabolomic analysis and IPA may represent a promising tool with which to explore and better understand both heart disease and TCM therapy.

## Introduction

Doxorubicin-induced cardiomyopathy (DOX-CM) is a severe complication of doxorubicin (DOX) treatment, a commonly used chemotherapeutic agent [1,2]. Characterized by cumulative and irreversible myocardial damage, the pathogenesis has not been fully elucidated. Previous studies determined that DOX-CM was associated with free radical damage, calcium overload, iron metabolism abnormality, DNA damage and cell apoptosis induced by DOX [1,3,4]. However, most of these studies focused on specific biochemical or signaling pathways; additional research studies of a more comprehensive nature are scarce.

Metabolomics is the latest developed “omics,” following genomics and proteomics. It studies both biological activity and gene regulation via an analysis of endogenous global metabolites found in both biological fluids and tissues [5–7]. It offers advantages such as high throughput detection in a limited sampling time, and may therefore allow researchers to explore changes in both biological activity and drug effects, which may represent a novel means by which to investigate the pathogenesis of DOX-CM [8–10].

Tan *et al*. used gas chromatography/mass spectrometry (GC/MS) based metabolomic technology to detect myocardial metabolic changes in mice at 72 hours following a single dose of DOX [11]. They identified 24 metabolites related to disturbances in myocardial energy metabolism. However, these metabolites were apparently associated with the acute phase of DOX treatment, during which heart structure and function do not change significantly. Therefore, the metabolic characteristics of DOX-CM remain unknown.

Shengmai Injection (SMI) is based on a famous Traditional Chinese Medicine (TCM), “Shengmai San (SMS),” which is officially recorded in Chinese Pharmacopoeia (version 2010) and has long been used to treat heart failure in China [12–14]. SMI consists of *ginseng*, *radix ophiopogonis* and *schisandra chinensis*, and may alleviate myocardial injury and heart dysfunction among patients treated with DOX and epirubicin (EPI) [15–17]. In rats with DOX-CM, it has been proven to exert cardioprotective effects via the inhibition of both myocardial oxidative stress and cardiomyocyte apoptosis [18,19]. However, as a result of its multi-component and multi-target nature and comprehensive regulation, the pharmacological mechanisms underlying the effects of SMI remain obscure. The emerging field of metabolomics matches well with the holistic concept of TCM, which treats diseases via the restoration of body homeostasis, and represents a promising potential approach with which to explore the effects and pharmacological mechanisms of TCM including SMI [20–22].

In the present study, we investigated cardiac metabolic characteristics in rats with DOX-CM, as well as the therapeutic effects of SMI, using a metabolomic approach based on GC/MS and ultra-performance liquid chromatography/tandem mass spectrometry (UPLC/MS/MS). Furthermore, we performed a bioinformatics analysis via Ingenuity Pathway Analysis (IPA) to explore the possible pathogenesis of DOX-CM and the pharmacological mechanisms underlying SMI’s actions.

## Methods and Materials

### Ethics Statement

All experimental procedures were approved by the Institutional Authority for Laboratory Animal Care of Xin Hua Hospital, Affiliated to Shanghai Jiao Tong University School of Medicine (Approval No. XHEC-F-2013-001), and conformed to the Guide for the Care and Use of Laboratory Animals, which was published by the National Institutes of Health (NIH publication, Eighth Edition, 2011).

### Animals

Sixty male Sprague-Dawley rats (250±11 g, eight weeks old) were purchased from SLAC Laboratories (Shanghai, China). All rats were housed under appropriate humidity (50–60%) and temperature (20–25°C), exposed to a 12-hour light and dark cycle, and fed standard chow and tap water ad libitum. The cages were kept dry and clean. The rats were randomized into a control group (n = 15), a DOX group (n = 15), an SMI group (n = 15) and a DOX + SMI group (n = 15). DOX (Sigma, Saint Louis, USA) was injected intraperitoneally (i.p.) via six equal injections (each containing 2.5 mg/kg DOX) within a period of two weeks, as described previously [23]. SMI (Hehuang, Shanghai, China) was injected i.p. via 12 equal injections (each containing the recommended 3 ml/kg dose of SMI) within a period of four weeks. In the DOX+SMI group, DOX was injected over a period of two weeks. SMI was injected one day before each DOX administration during the two weeks in question but was administered alone during the following two weeks. The control group received isometric saline. The rats were monitored three times a day during an eight-week follow-up period. The following steps were taken to minimize the suffering of the rats. First, the rats were handled gently to reduce their discomfort and distress. Second, non-invasive echocardiography was utilized to assess the rats’ heart structures and function, as opposed to invasive hemodynamic testing. Third, anesthesia was administered prior to blood sample collection, echocardiography and animal sacrifice. Additionally, drug administration, anesthesia, testing and animal sacrifice were undertaken in separate rooms to avoid instilling fear in the other rats. Furthermore, the rats were treated humanely to minimize their suffering. Rats exhibiting symptoms of moribundity were euthanized. The signs and symptoms of moribundity were as follows: (a) impaired ambulation (unable to reach either food or water easily); (b) any obvious severe illness, including signs and symptoms such as lethargy (drowsiness, aversion to activity, a lack of physical or mental alertness), anorexia (loss of appetite, particularly prolonged behavior), bleeding, difficulty breathing, or chronic diarrhea; (c) an inability to remain upright; (d) either rapid weight loss or a net weight loss of more than 20% of body weight; and (e) unconsciousness or unresponsiveness to external stimuli.

### Serum assay

Brain natriuretic peptide (BNP), a biomarker of congestive heart failure, is clinically used to assess the severity of heart dysfunction. Cardiac troponin I (cTNI), myoglobin and creatine kinase-MB (CK-MB) are biomarkers of myocardial injury. At the end of the follow-up period, the rats were anesthetized using chloral hydrate (300 mg/kg, i.p.), and their body weights were measured. They were subsequently placed on a warm platform. Blood samples were collected via the tail vein and centrifuged to obtain serum. The latter was stored at -80°C until needed. The serum concentrations of BNP, cTNI, myoglobin and CK-MB were detected using ELISA kits in accordance with the manufacturers’ instructions (BNP Elisa Kit: Sigma, Saint Louis, USA; cTNI Elisa Kit: Guduo, Shanghai, China; Myoglobin Elisa Kit: Xinyu, Shanghai, China; CK-MB Elisa Kit: Uscnk, Wuhan, China).

### Echocardiography

Following blood sample collection, transthoracic echocardiography was performed using a Vevo770 High-Resolution Micro-Ultrasound System (VisualSonics, Toronto, Canada), with a cardiac probe (RMV716). The rats were placed in the supine position after both the anterior and left lateral thoracic regions were shaved. Ultrasound transonic gel was placed on the thorax to optimize visibility. M-mode and two-dimensional echocardiographic images were recorded at the level of the papillary muscles. Left ventricular (LV) internal dimensions (LVID), LV anterior wall thickness (LVAW) and LV posterior wall thickness (LVPW) were measured during both systole and diastole. The ejection fraction (EF), fractional shortening (FS), LV volume at end-systole (LVESV) and end-diastole (LVEDV) were calculated using the above parameters.

### Myocardium sample collection

Following echocardiography, the anaesthetized rats were euthanized according to the AVMA Guidelines for the Euthanasia of Animals (2013 Edition). Specifically, the unconscious rats were rapidly injected with potassium chloride (2 mmol/kg). Following the onset of cardiac arrest, the animals’ hearts were harvested and weighed. The heart weight / body weight ratio was calculated. Portions of the ventricular myocardium were subsequently frozen using liquid nitrogen and kept at -80°C until needed for Western blotting and metabolomic analysis. The other samples were fixed with formalin for the Masson assay.

### Masson assay

A Masson assay was utilized to detect fibrosis within the cardiac tissue samples. Fresh myocardial tissues were fixed in 10% buffered formalin, embedded in paraffin, and cut into 5-μm thick sections. The sections were then stained with Masson’s trichrome and observed under a microscope. Eight sights within each section were randomly selected; the fibrotic area within each section was measured and analyzed using a HMIAS Series Color Medical Image Analyze System (Champion Image Ltd., China).

### Western blotting

Protein was extracted from cardiac tissue homogenates with ice-cold RIPA buffer and protease inhibitors (Roche, Basel, Switzerland). Protein concentrations were determined via a Bradford protein assay (Beyotime, Nantong, China). Equal amounts (30–50 μg) of protein were separated via 10% sodium dodecyl sulfate-polyacrylamide gel electrophoresis (SDS-PAGE) and transferred onto a polyvinylidene fluoride (PVDF) membrane (Millipore, Billerica, MA, USA). After being blocked with 5% skim milk in Triethanolamine-Buffered Saline Solution with Tween (TBST), the membranes were incubated with a 1:1000 dilution of primary antibodies against Bax, Bcl-2 and caspase-3 (Cell Signaling Technology, Danvers, Massachusetts, USA) at 4°C overnight. Following three washes in TBST, the membranes were incubated with horseradish peroxidase (HRP)-conjugated secondary antibodies (Cell Signaling Technology, Danvers, Massachusetts, USA) at a 1:1000 dilution at room temperature for 1 hour. Following three washes in TBST, the protein bands were detected using enhanced chemiluminescence (ECL) reagents (Millipore, Billerica, MA) and quantified using the ChemiDoc XRS imaging system (Bio-Rad, Hercules, CA, USA).

### Metabolic profiling

Frozen myocardial samples from each of the groups were submitted to Metabolon Inc. (Durham, NC, USA) for sample extraction and metabolomic analysis. Global metabolic profiling was performed via a combination of two independent platforms, GC/MS and UPLC-MS/MS. The detailed descriptions of the platforms, including instrumentation configurations, data acquisition and processing, compound identification and quantitation, have been described previously [24–26]. Briefly, the myocardial samples were extracted using methanol based solutions. The resulting extract was divided into the following two fractions: one for analysis via GC/MS and another for analysis via LC/MS.

For the GC/MS analysis, the extract was derivatized under dried nitrogen using bistrimethyl-silyl-triflouroacetamide. The GC column was 5% phenyl, and the temperature ramp was from 40 to 300°C within a 16-minute period. The samples were analyzed on a Thermo-Finnigan Trace DSQ fast-scanning single-quadrupole mass spectrometer using electron impact ionization.

The UPLC-MS/MS platform was based on a Waters ACQUITY UPLC and a Thermo-Finnigan LTQ mass spectrometer, including an electrospray ionization source and a linear ion-trap mass analyzer. The extract was split into two aliquots and reconstituted in both acidic and basic LC-compatible solvents. One aliquot was analyzed using acidic positive ion optimized conditions, and the other, using basic negative ion optimized conditions in two independent injections, using separate dedicated columns. The MS analysis alternated between MS and data-dependent MS/MS scans using dynamic exclusion.

The biochemical molecules detected in the samples were identified via automated comparisons to Metabolon’s reference library entries, including molecular ions, retention time, and MS/MS patterns of thousands of commercially available purified standard biochemicals tested using Metabolon’s mass spectrometry platform. The combination of chromatographic properties and mass spectra gave an indication of a match to a specific metabolite.

### Statistical data processing and analysis

The results of the weight, echocardiography and cardiac biomarker, fibrosis and apoptosis assays were presented as means ± SDs. The original metabolomic data were scaled via division with a median value after replacing missing values with the minimum observed values. The differences between any two groups were analyzed via independent t-tests, using SPSS 17.0 software (SPSS Inc., Chicago, USA). A *p*≤ A SS Inc., Chicago,statistically significant, and 0.05<*p*<0.10 was considered a modest difference. A principle component analysis (PCA) analysis was performed with the program “R” (version 2.15). A bioinformatics analysis was performed to investigate the interactions among differentially expressed metabolites and the possible pathogenesis of DOX-CM, as well as the pharmacological mechanisms of SMI, using IPA software (Ingenuity, Redwood City, USA), a web-based software that helps researchers to analyze, understand, and visualize the interplay among genes, proteins, metabolites, drugs, and normal cellular and disease processes [27].

## Results

### General toxicity and heart weight

**Fig 1:**
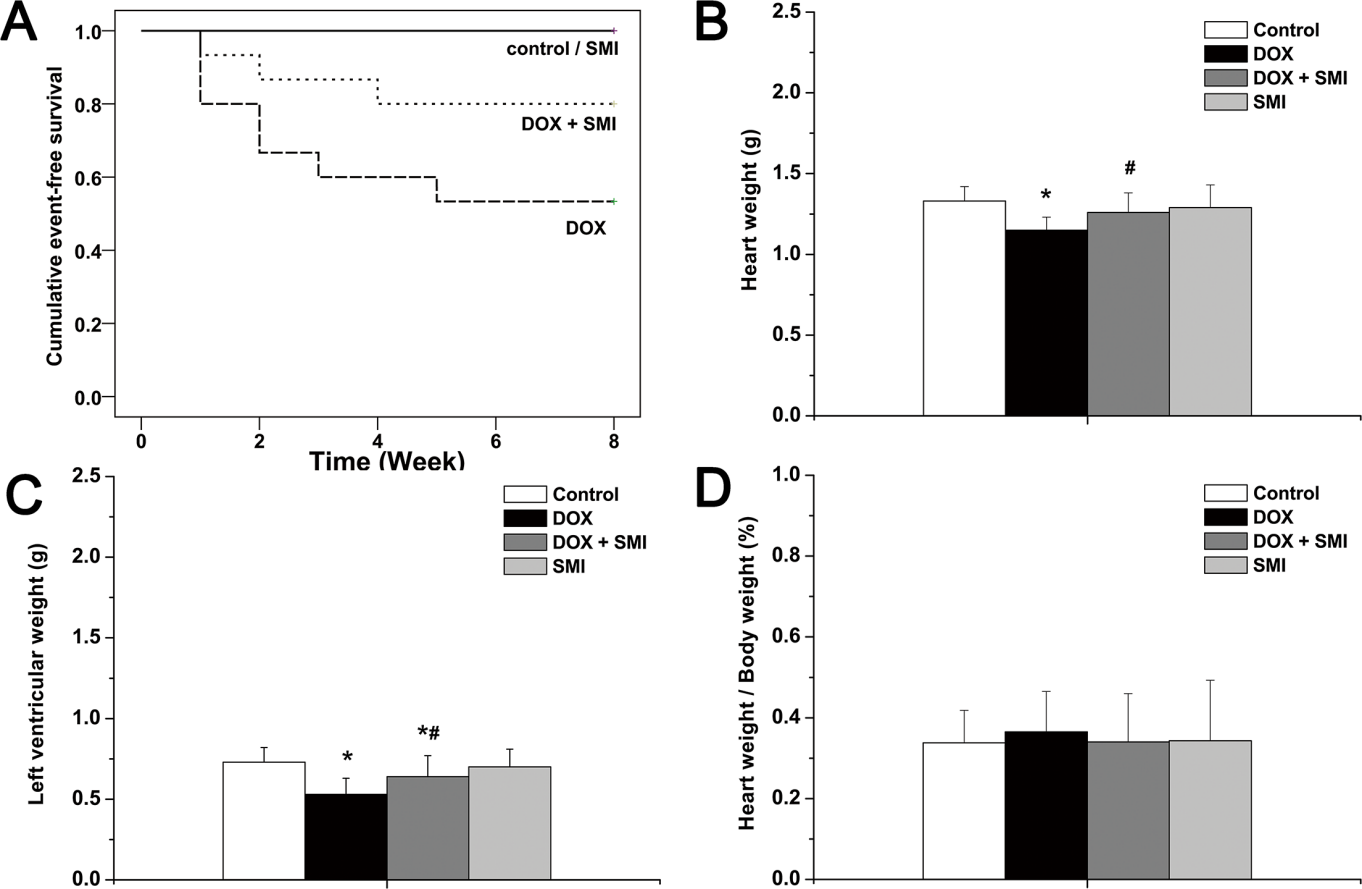
General appearance across all groups. a survival; b heart weight; left ventricular weight; d heart weight / body weight. Compared with the control group, *p<0.01; compared with the DOX group, ^#^ *p*<0.05.

At the end of the eight-week follow-up period, none of the rats in either the control group or the SMI group died. Eight rats (53.3%) in the DOX group survived, and 12 rats (80%) in the DOX + SMI group survived. The survival rate of the DOX group was significantly higher than that of the control group (*p*<0.01). Compared with the DOX group, the survival rate of the DOX + SMI group increased but did not reach statistical significance at this time point (*p* = 0.12) (Fig 1A). The heart weight and LV weight of the DOX group were both significantly lower than the corresponding values of the other groups (*p*<0.01, Fig 1B and 1C), whereas the heart weight / body weight ratio was slightly higher compared with the other groups (*p*>0.05, Fig 1D).

### Myocardial biomarkers

**Fig 2:**
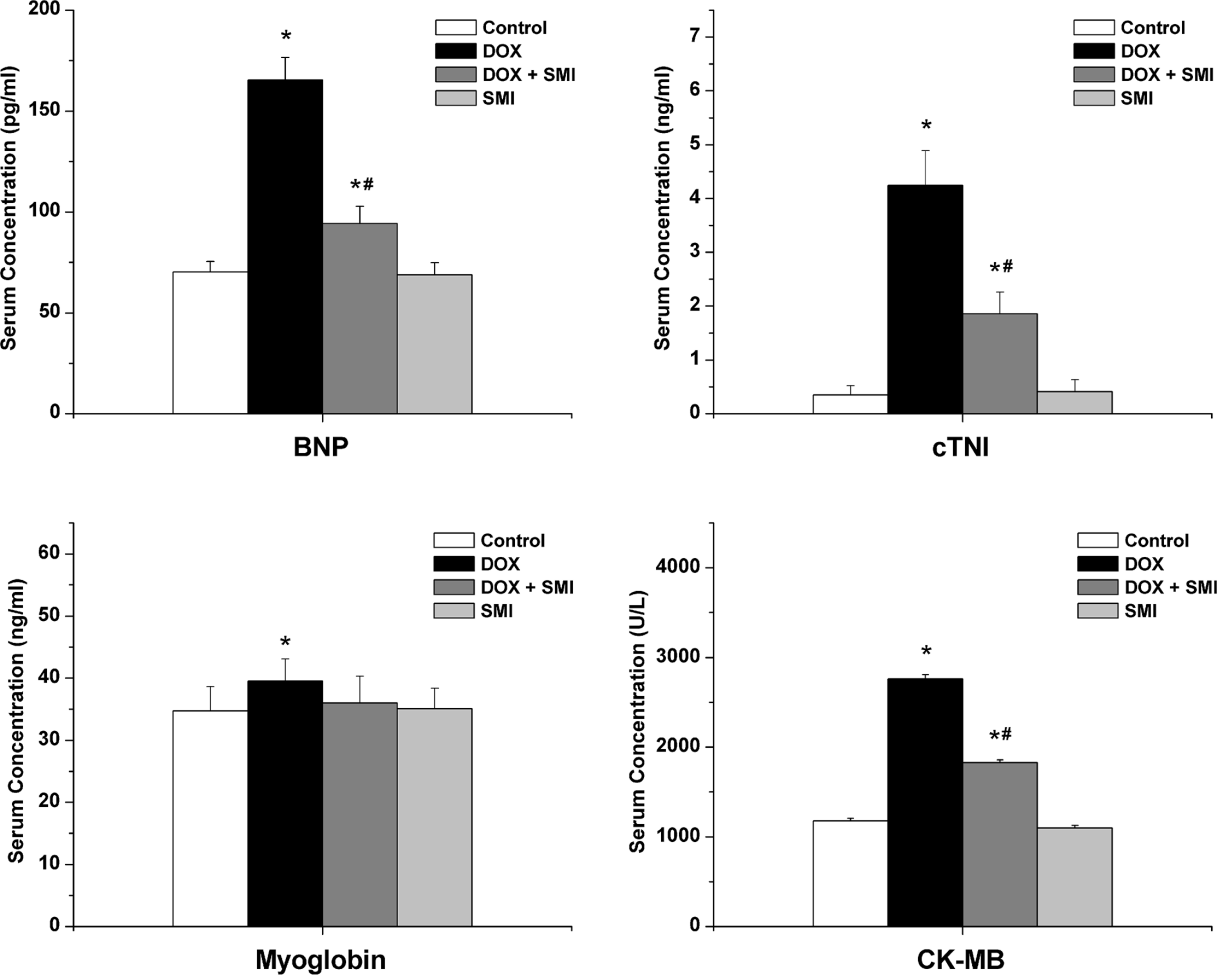
The serum concentrations of the cardiac biomarkers across all groups. Compared with the control group, *p<0.05; compared with the DOX group, ^#^ *p*<0.01.

The serum concentrations of the cardiac biomarkers are included in Fig 2. A biomarker of congestive heart failure, the serum BNP concentration in the DOX group was significantly higher compared with the other groups at the end of eight weeks (*p*<0.01). The serum BNP concentration in the DOX + SMI group was significantly lower compared with the DOX group (*p*<0.01). The serum concentrations of cTNI, myoglobin and CK-MB of the DOX group were significantly higher than those of the control group (*p*<0.05), whereas the concentrations of the DOX + SMI group were significantly lower than those of the DOX group, with the exception of myoglobin (*p*<0.01). The myoglobin concentration of the DOX + SMI group was mildly lower than that of the DOX group (*p* = 0.07).

### Echocardiography

**Fig 3:**
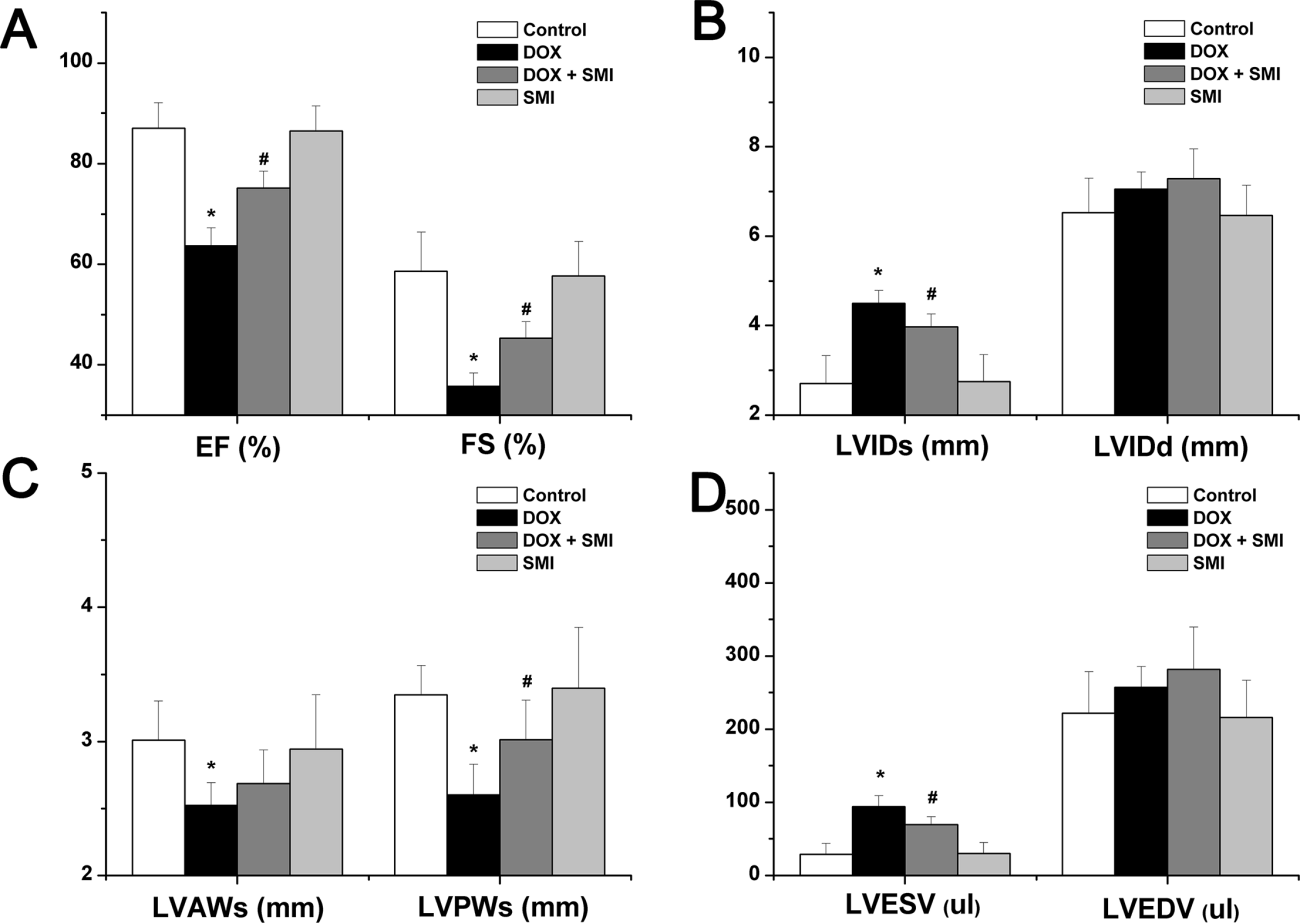
The echocardiographic parameters across all groups. a EF and FS; b LVIDs and LVIDd; c LVAWs and LVPWs; d LVESV and LVEDV. Compared with the control group, *p<0.01; compared with the DOX group, ^#^ *p*<0.01.

The changes in heart structure and function detected via echocardiography are included in both Fig 3 and S1 Table. Briefly, in the DOX group, LVIDs and LVESV increased significantly (*p*<0.01), whereas LVAWs, LVPWs, EF and FS decreased significantly compared with the control group (*p*<0.01). In the DOX + SMI group, LVIDs, LVPWs, LVESV, EF and FS each improved significantly compared with the DOX group (*p*<0.01).

### Myocardial fibrosis

**Fig 4:**
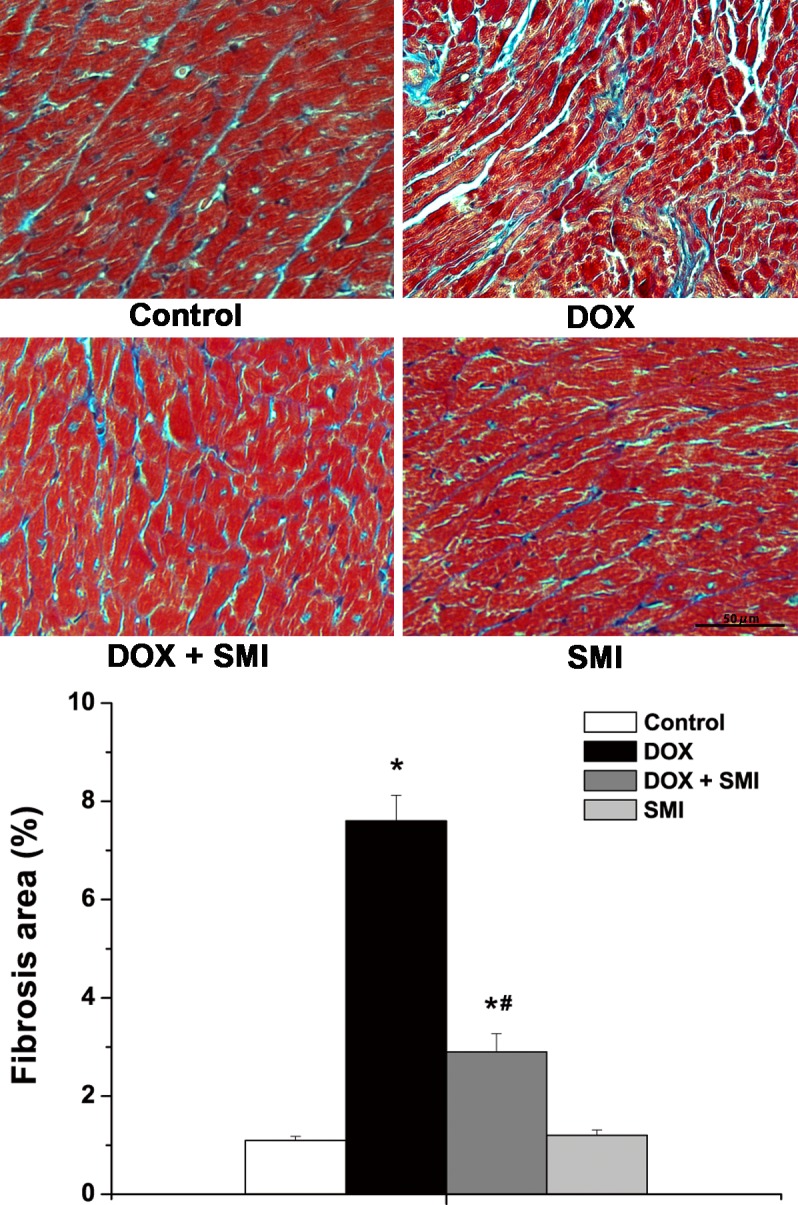
Myocardial fibrosis via the Masson assay. Compared with the control group, *p<0.01; compared with the DOX group, ^#^ *p*<0.01.

The Masson assay demonstrated that DOX induced remarkable fibrosis in the myocardial tissues of rats, whereas SMI significantly decreased cardiac fibrotic area. As demonstrated in Fig 4, the ratio of fibrosis area in the DOX group was 7.6±0.52%, which was significantly higher than that of the control group (1.1±0.08%, *p*<0.05). In the DOX + SMI group, the fibrotic ratio was 2.9±0.37%, which was significantly lower than that of the DOX group (*p*<0.05).

### Myocardial apoptosis

**Fig 5:**
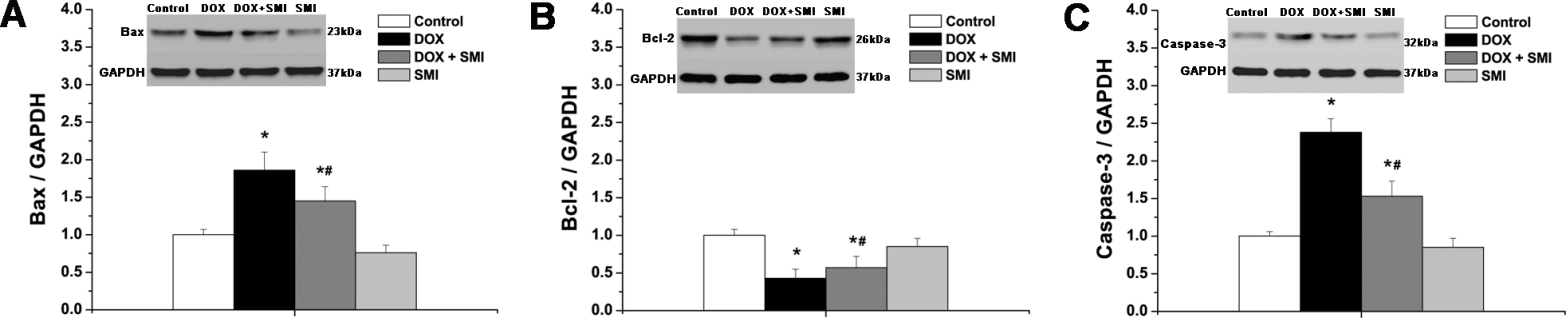
The expression of the apoptosis related proteins across all groups. a Bax; b Bcl-2; c caspase-3. Compared with the control group, *p<0.01; compared with the DOX group, ^#^ *p*<0.05.

The myocardial expression levels of the apoptosis related proteins (Bax, Bcl-2 and caspase-3) across all groups are included in Fig 5. The expression levels of both Bax and caspase-3 were significantly higher in the DOX group compared with the control group (*p*<0.05), whereas anti-apoptotic Bcl-2 expression was significantly lower in the DOX group compared with the control group (*p*<0.05). Moreover, the myocardial expression levels of both Bax and caspase-3 in the DOX + SMI group were significantly lower than those of the DOX group (*p*<0.05), whereas Bcl-2 expression was higher compared with the DOX group (*p*<0.05).

### Overview of cardiac metabolic profiling

**Fig 6:**
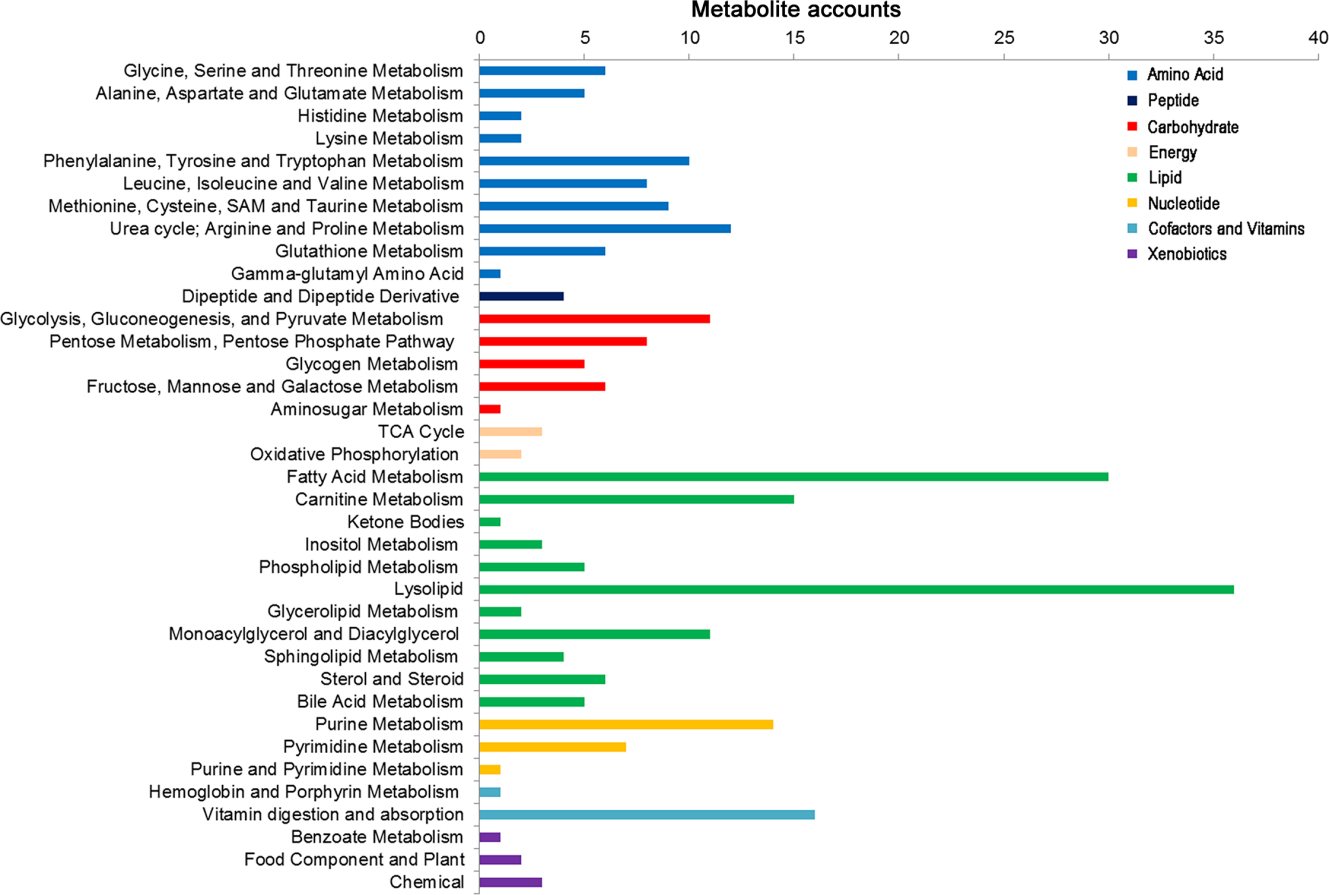
The metabolic pathways of 264 metabolites across all groups.

**Fig 7:**
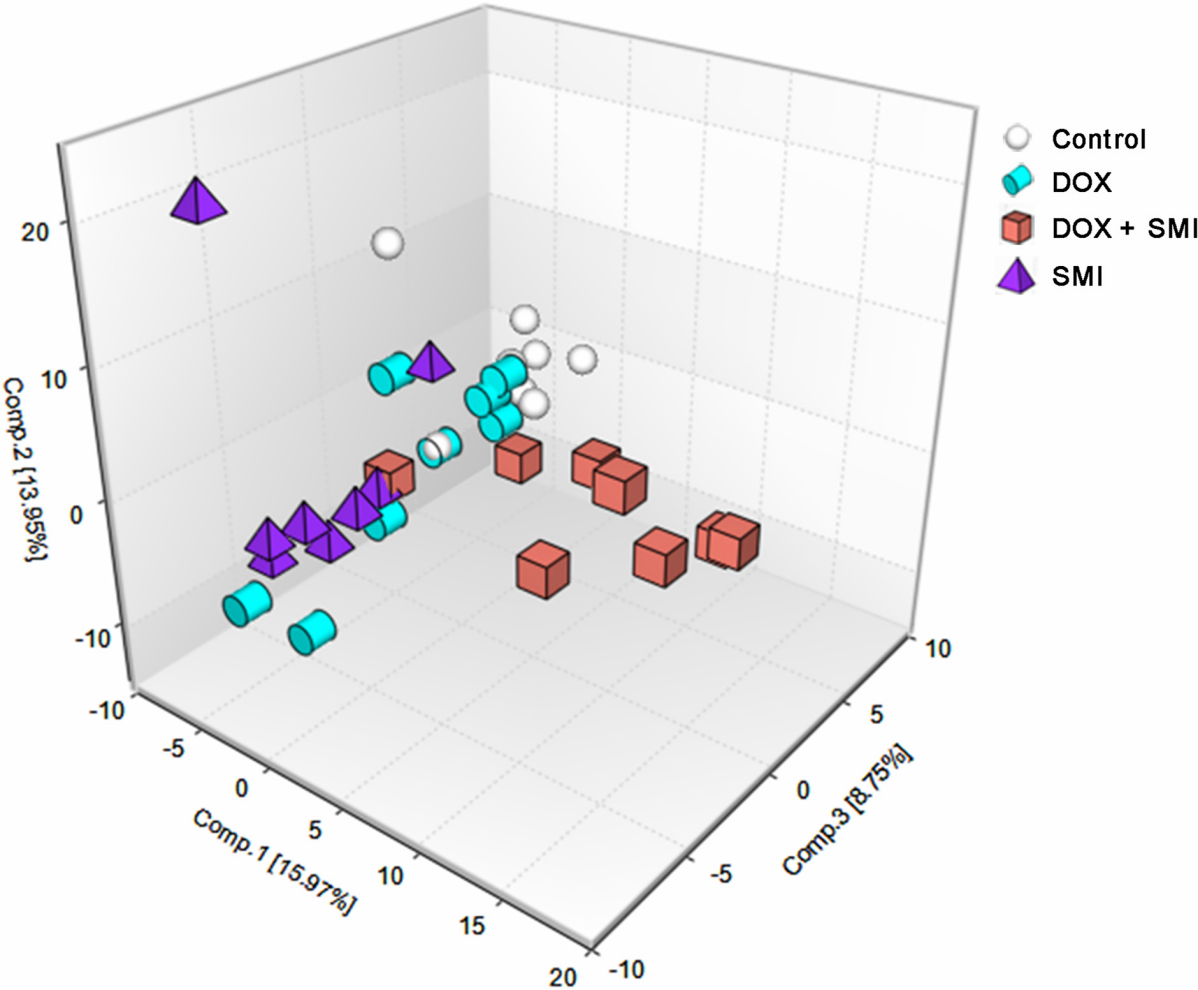
A PCA score plot generated from 264 metabolites across all groups.

A total of 264 metabolites of known identity were detected across all of the groups, which were mapped to eight super-pathways and 66 sub-pathways, according to the database from the Kyoto Encyclopedia of Genes and Genomes (KEGG) and the Human Metabolome Database (HMDB) (Fig 6 and S2 Table). These super-pathways have been linked to the metabolism of amino acids, peptides, carbohydrates, energy, lipids, nucleotides, cofactors/vitamins and xenobiotics, covering each of the central metabolic pathways. The PCA demonstrated a distinct separation across the four groups, suggesting the existence of different metabolic characteristics among the treatments utilized (Fig 7).

### Metabolic profiling of the rats with DOX-CM

**Fig 8:**
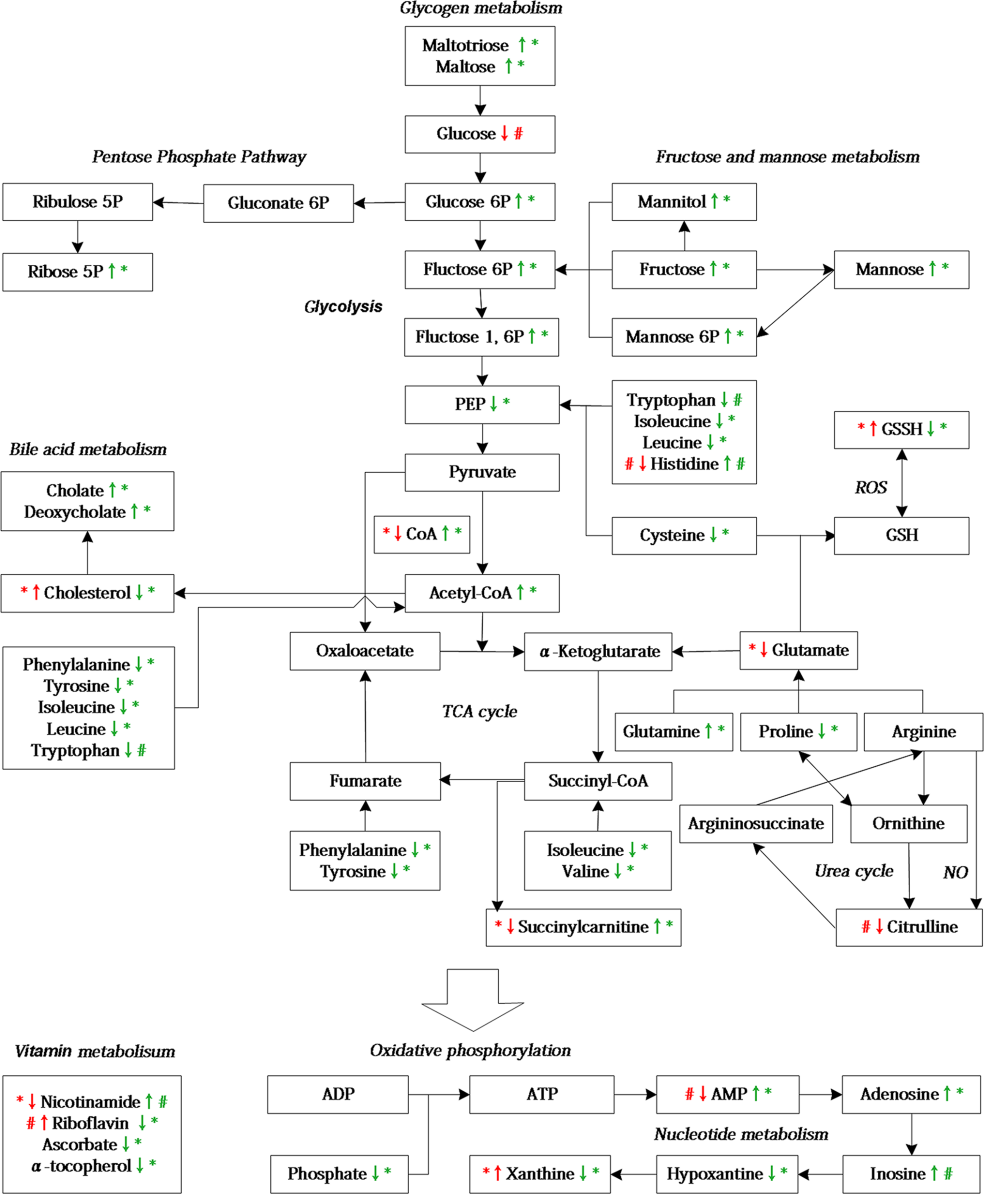
A simplified schematic diagram of the metabolic changes induced by DOX-CM and SMI treatment. The red arrows represent DOX group versus the control group; the green arrows represent the DOX + SMI group versus the DOX group, *p<0.05, 0.05<^#^ *p*<0.10.

**Fig 9:**
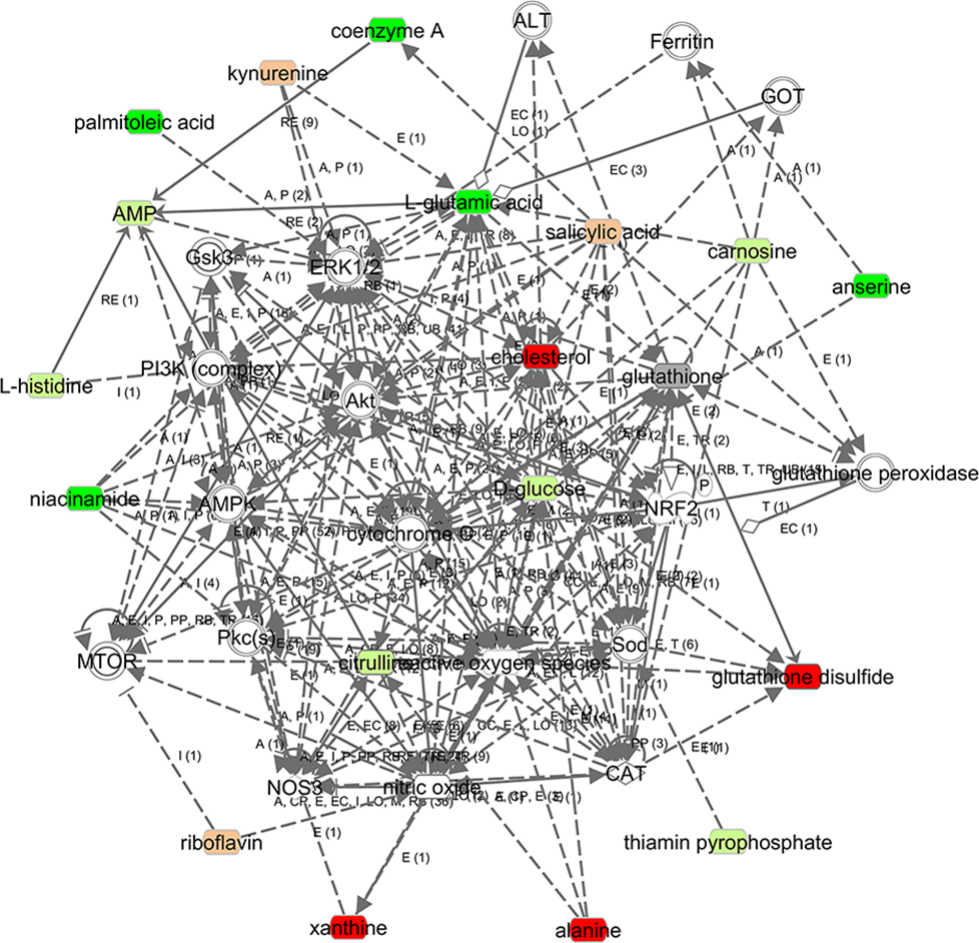
The molecular network of DOX-CM via IPA. The metabolites are represented as colored nodes. The red nodes represent the significantly upregulated metabolites; the light red nodes represent the modestly upregulated metabolites; the green nodes represent the significantly downregulated metabolites; the light green nodes represent the modestly downregulated metabolites, and the gray nodes represent the unchanged metabolites. The transparent entries were molecules from the Ingenuity Knowledge Database. The biological relationship between two nodes is represented as a line. A solid line indicates a direct physical relationship between the corresponding molecules, whereas a dotted line indicates an indirect functional relationship.

Compared with the control group, 31 significantly altered metabolites and 19 modestly altered metabolites were observed in the rats with DOX-CM (S3 Table). Among them, 24 metabolites were upregulated, whereas the others were downregulated. These metabolites were primary involved in lipid, amino acid, vitamin and energy metabolism. A simplified schematic diagram of these metabolites is included in Fig 8. To further investigate the interactions among the altered metabolites and explore the possible pathogenesis of DOX-CM, a bioinformatics analysis was performed via an IPA, and a biological network was built by combining the metabolites and the pathways, in which important signaling molecules such as PI3K, AKT, AMPK, ERK, AKC, mTOR and NRF2, as well as enzymes such as SOD, GPX, CAT, GOT, ALT and eNOS, were included (Fig 9).

### Metabolic profiling of the SMI treatment of rats with DOX-CM

**Fig 10:**
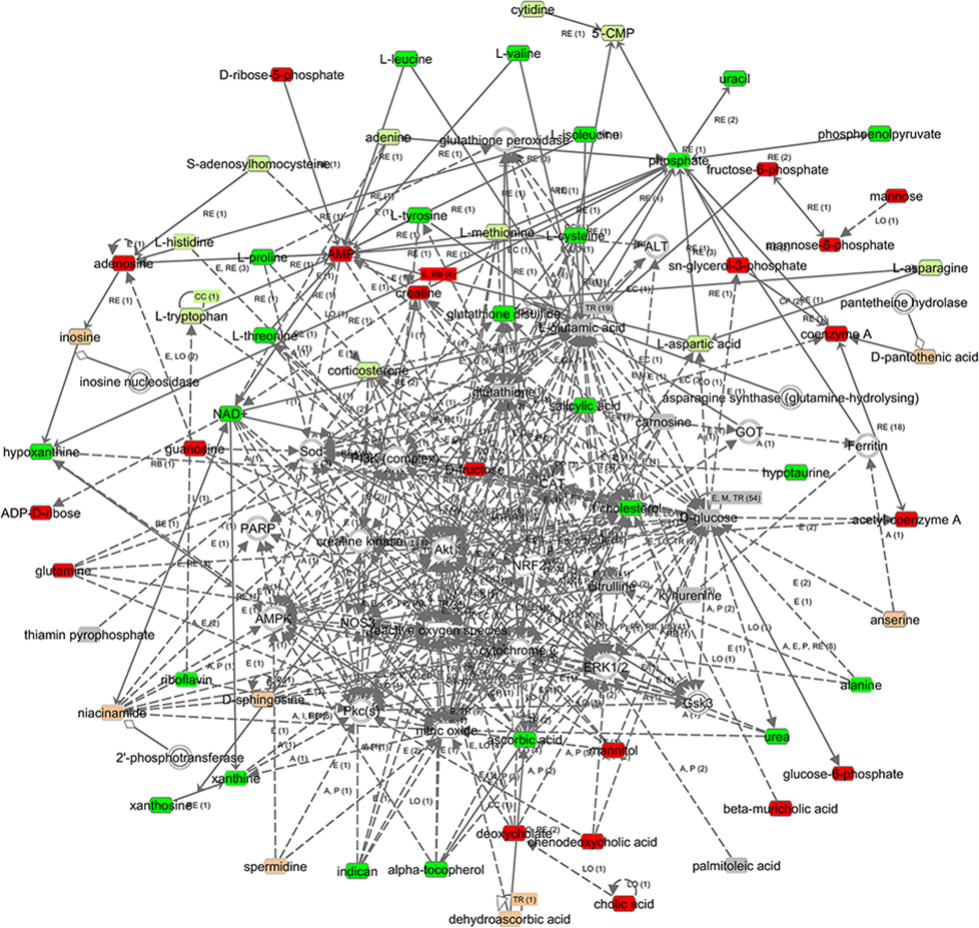
The molecular network of SMI treatment via IPA. The metabolites are represented as colored nodes. The red nodes represent the significantly upregulated metabolites; the light red nodes represent the modestly upregulated metabolites; the green nodes represent the significantly downregulated metabolites; the light green nodes represent the modestly downregulated metabolites, and the gray nodes represent the unchanged metabolites. The transparent entries were molecules from the Ingenuity Knowledge Database. The biological relationship between two nodes is represented as a line. A solid line indicates a direct physical relationship between the corresponding molecules, whereas a dotted line indicates an indirect functional relationship.

Compared with the DOX group, 82 significantly altered metabolites and 25 modestly altered metabolites were observed in the rats with DOX-CM treated with SMI (S4 Table). Among them, 52 metabolites were upregulated, whereas the others were downregulated. Some of the altered metabolites in the DOX group, such as oxidized glutathione (GSSH) and cholesterol, were either reversed or attenuated via SMI treatment. The simplified schematic diagram of these metabolites is included in Fig 8. The integrated biological network of SMI treatment depicted via the IPA demonstrated more altered metabolites and possible interactions compared with the DOX action network (Fig 10).

## Discussion

In rats with DOX-CM, a significant increase in serum cTNI, myoglobin and CK-MB, and increased caspase-3 and BAX to Bcl2 ratio were strongly suggestive of ongoing necrotic and apoptotic cell death. Moreover, remarkable cardiac remodeling and heart dysfunction occurred, as evidenced by significantly increased cardiac fibrosis and BNP levels, as well as diminished echocardiographic features. Furthermore, metabolic perturbations occurred in the rats with DOX-CM. The metabolomic analysis detected 264 metabolites associated with carbohydrate and energy metabolism, lipid metabolism, cofactor and vitamin metabolism, and amino acid and nucleotide metabolism. The metabolic characteristics were clearly distinguished across each of the different groups. The IPA demonstrated that important biochemical molecules such as AKT, AMPK and SOD were involved in the biological network associated with DOX-CM. Moreover, SMI attenuated the metabolic dysfunction caused by DOX and improved the myocardial injury and the heart structure and function of the rats with DOX-CM.

Cardiac energy metabolism undergoes remodeling in the setting of chronic heart failure. The oxidation of fatty acids was inhibited, whereas the utilization of glucose was increased [28,29]. Accordingly, using the isolated perfused hearts of rats with DOX-CM, Carvalho *et al*. determined that energy substrates shifted from the preferential oxidation of fatty acids to the enhanced oxidation of glucose [30]. In accordance with these findings, cardiac glucose levels were modestly decreased in the DOX group of our study, suggesting the presence of an energy deficiency in the DOX-CM rats and the increased consumption of glucose. In the DOX + SMI group, the accumulation of multiple glycogen metabolites, including maltohexaose, maltopentaose, maltotetraose, maltotriose and maltose, may have been reflective of the catabolism of glycogen as a means of replenishing glucose, as glycogen is an immediate source of glucose that cardiac tissue may be utilized to maintain metabolic homeostasis. Moreover, the SMI-treated tissues possessed higher levels of the glycolytic intermediates glucose 6-phosphate (G6P) and fructose 6-phosphate (F6P), and a significantly diminished level of phosphoenolpyruvate (PEP), which may be indicative of altered glycolysis. The elevated G6P and F6P levels may increase substrate availability for the activated pentose phosphate pathway (PPP), as demonstrated by the elevated ribose-5-phosphate level. The activated PPP facilitates NADPH regeneration for anabolic growth and glutathione (GSH) reduction, and reportedly sustains cardiac progenitor cell availability in the setting of diabetes [31]. Another study demonstrated that the pharmacological activation of the PPP alleviated oxidative stress and prevented the cell death associated with congestive heart failure [32]. Additionally, mitochondrial metabolism may also be impacted, as the tricarboxylic acid (TCA) cycle intermediate succinylcarnitine (surrogate for succinyl-CoA) differed significantly between the groups as demonstrated below. Succinylcarnitine declined significantly in the DOX group but increased in the DOX + SMI group, which suggests that SMI attenuated the disruption of the TCA caused by DOX.

Lipid metabolism is disrupted in the setting of DOX-CM [33,34]. Cholesterol increased significantly in the DOX group, which may have been the result of a lipolysis blockade caused by DOX [11,35]. In the DOX + SMI group, the cholesterol level declined with increases in the levels of bile acids such as cholate and deoxycholate, which may indicate that SMI improved cholesterol metabolism by increasing bile acid synthesis. Yao *et al*. observed that SMS lowered hepatic cholesterol and triglyceride levels and promoted bile acid excretion in rats receiving a high-cholesterol diet, which supported our findings to some extent [36].

Amino acids are important alternative energy substrates that may be transformed into intermediates of both glycolysis and the TCA, such as PEP and acetyl-CoA [37]. In the DOX group, the decreased glutamate and histidine levels were used to attenuate the ATP depletion caused by DOX. In the DOX + SMI group, valine, cysteine, phenylalanine, tyrosine, leucine and isoleucine decreased significantly, which suggests that SMI promoted the use of both glucogenic and ketogenic amino acids.

Oxidative stress is a key mechanism of DOX-CM [3,38]. Oxygen radicals (ROS) induced by DOX triggers the formation of a disulfide bond with another oxidized molecule of GSH to form GSSH [39]. The GSSH level increased significantly in the DOX group, whereas it was decreased in the DOX + SMI group, suggesting that SMI scavenged the ROS produced by DOX. Notably, cysteine (the rate limiting metabolite in GSH biogenesis) and hypotaurine (produced via the reduction of excess cysteine) levels were decreased in the DOX + SMI group, suggesting that cysteine was utilized to synthesize GSH for free radical detoxification. Moreover, the elevated ophthalmate level noted in the DOX + SMI group may be indicative of cystathionine degradation as a means of supporting cysteine biogenesis to replenish GSH. Therefore, SMI may be beneficial for GSH maintenance.

Citrulline is made from ornithine in the urea cycle, or from arginine, as a by-product of nitric oxide (NO) formation. The latter is a potent cardioprotective molecule that exerts multiple effects, including the inhibition of oxidative stress [40,41]. A previous study reported that NO levels were decreased in the setting of DOX-induced congestive heart failure [42]. Therefore, the modest decrease in citrulline levels noted in DOX group may have been reflective of a low level of NO in the setting of DOX-CM.

Xanthine, an intermediate in the degradation of adenosine monophosphate (AMP), plays an important role in the formation of ROS [43]. In the DOX group, the observed significant increase in the level of xanthine may have been reflective of oxidative stress. In the DOX + SMI group, the levels of xanthine and its precursor, hypoxanthine, decreased significantly; however, their upstream molecules, including inosine, adenosine and AMP, increased, suggesting that SMI inhibited the activity of inosine ribohydrolase, a key enzyme that catalyzes the conversion of inosine to hypoxanthine.

The levels of the multiple vitamins were altered following different treatments, including riboflavin, nicotinamide, ascorbate and α-tocopherol. As a naturally occurring antioxidant, riboflavin decreases oxidative damage [44]. In the DOX group, the noted modest increase in riboflavin may have been a compensatory response to oxidative stress. In contrast to riboflavin, nicotinamide, another antioxidant, decreased significantly in the DOX group. Nicotinamide improved left ventricular function by attenuating oxidative stress-mediated myocardial remodeling [45]. Therefore, the reduced nicotinamide may participate in the antioxidative response in DOX-CM. In the DOX + SMI group, the restoration of both riboflavin and nicotinamide may have been reflective of the attenuation of oxidative stress. Additionally, the DOX + SMI group exhibited significantly depleted antioxidants, ascorbate and α-tocopherol, suggesting that SMI promoted the utilization of ascorbate and α-tocopherol for antioxidation. Collectively, these findings suggest that vitamins play vital roles in attenuating the oxidative stress induced by DOX and that SMI protects heart tissue via the modulation of vitamin metabolism.

To further explore the interactions among the altered metabolites and the possible pathogenesis of DOX-CM, a biological network was built via an IPA, a network in which several important signaling molecules and enzymes were involved (Fig 9). AKT was a major hub molecule in this network. PI3K-AKT is activated by other signaling molecules such as mTOR and PKC [46,47]. On another hand, AKT participates in downstream multi-pathways. For example, as demonstrated in Fig 9, AKT influences downstream ERK1/2 and GSK3β, which promotes both the phosphorylation and the nuclear translocation of NRF2 and results in the activation of antioxidases such as SOD, GPX, CAT and GST [48–50]. Previous studies have proven that the dephosphorylation of both AKT and ERK1/2 by DOX was related to myocardial injury, whereas the phosphorylation of both AKT and ERK1/2 by dexrazoxane ameliorates DOX-induced cardiotoxicity [51,52]. Some antioxidases, including SOD, GPX, CAT and GST, were reportedly inhibited by DOX in previous studies [53–55]. Given the above biological network and experimental evidence, we hypothesized that DOX induces cardiac oxidative insults partially by the inactivation of antioxidases via the inhibition of AKT-ERK1/2-NRF2 signaling. However, this pathway and its relationships with surrounding metabolites in the network must be explored further.

AMPK is an additional major hub molecule. The activation of AMPK promotes glucose uptake, glycolysis, fatty acid oxidation and mitochondria biogenesis. Moreover, AMPK plays an important role in cellular redox balance [56,57]. Cheng *et al*. reported that the activation of AMPK by resveratrol inhibited the formation of ROS induced by NADPH oxidase [58]. Silencing AMPKα1 reportedly decreases the expression of SOD, CAT and gamma-glutamylcysteine synthase in endothelial cells [59]. Wang *et al*. observed that DOX inhibited AMPK signaling in mouse embryonic fibroblasts and cardiomyocytes [60]. Given the above biological network and experimental evidence, we speculated that DOX aggravated cellular energy deficits and oxidative stress via the inhibition of AMPK signaling, a hypothesis that warrants further investigation using molecular biology techniques.

Our study demonstrated that SMI improved cardiac function and structure, and attenuated metabolic derangements in rats with DOX-CM. The underlying pharmacological mechanisms may be multifold. First, SMI improved energy metabolism in the setting of DOX-CM by promoting glycogenolysis, glycolysis and the utilization of both glucogenic and ketogenic amino acids. Second, SMI regulated redox-related molecules, including GSSH, cysteine and ascorbate, suggesting that it exerts multiple antioxidative effects via GSH maintenance and vitamin absorption and utilization. Moreover, a recent study demonstrated that SMI protected against oxidative stress by increasing SOD expression and reducing iNOS expression [61]. Additionally, the biological network of SMI treatment demonstrated many possible interactions between the altered metabolites and the pathways, providing new clues regarding the pharmacological mechanisms of SMI, which warrant exploration.

In the DOX group of our study, 31 significantly altered metabolites and 19 modestly altered metabolites were detected, which may have been reflective of the chronic-phase effects of DOX and a concomitant state of heart failure. Most of these metabolites were different than those noted by Tan *et al*. [11], as their findings may have represented the effects of acute DOX cardiotoxicity. Among the 24 altered metabolites noted by their research study, only cholesterol and alanine exhibited changes similar to those noted in our study, whereas the others did not change significantly in our study, which may indicate that the acute metabolic changes caused by DOX were attenuated during the chronic phase.

There were several limitations to our study. First, although cardiac metabolism was the primary focus regarding the effects of DOX-CM, studying the accompanying metabolic changes in the serum, urine, feces, liver and kidney may be beneficial. However, due to funding limitations, we were unable to pursue these studies. Second, to comprehensively understand the metabolic characteristics of DOX-CM and SMI treatment, three metabolites approaching statistical significance (0.05<*p*<0.10) were carefully discussed above, specifically glucose, citrulline and histidine, providing new clues regarding future areas of study. However, the description of these metabolites should be viewed cautiously and warrants further exploration. Third, although the biological networks built by IPA exhibited many possible interactions among the included metabolites and signaling pathways, the present study could not draw any conclusions regarding said relationships; therefore, they must be studied further using molecular biology techniques. Fourth, the present study investigated the metabolic profiling of rats with DOX-CM. Due to the complexity of the observed metabolic changes, the integration of metabolomics with other “omics” such as proteomics and transcriptomics may be helpful in providing us with a more comprehensive and deeper understanding of DOX-CM. Finally, although SMI improved cardiac function and structure and attenuated the metabolic perturbations of the rats with DOX-CM, the increased survival rate did not reach statistical significance at the end of the eight-week follow-up period (*p* = 0.12). Studies with longer follow-up periods and larger sample sizes are necessary to further explore the effect of SMI on survival.

## Conclusions

There were significant cardiac metabolic disturbance noted in the rats with DOX-CM, which primarily involved lipid, amino acid, vitamin and energy metabolism, and may have been reflective of the energy disruption and oxidative stress induced by DOX. Furthermore, SMI may be a useful agent in treating the cardiac damage induced by DOX, as it may alleviate cardiac remodeling and improve both cardiac function and the metabolic perturbations of rats with DOX-CM. The possible mechanism underlying these effects may be related to the improvement of energy metabolism and the attenuation of oxidative stress. Moreover, the IPA demonstrated that several important signaling molecules and enzymes interacted with the altered metabolites, which may play important roles in both DOX-CM pathogenesis and the effects exerted by SMI treatment, and may provide new clues worth exploring in subsequent studies. These findings have expanded our knowledge regarding the metabolic characteristics of DOX-CM, provided new insights into DOX-CM pathogenesis and SMI effects, and suggested that the combination of metabolomic analysis and IPA may represent a promising tool with which to explore and better understand both heart disease and TCM therapy.

## Supporting Information

S1 ARRIVE Checklist NC3Rs ARRIVE Guidelines Checklist. (PDF)

S1 Table Echocardiography parameters. Compared with the control group, p*<0.01; compared with the DOX group, *p* ^#^<0.01.
(XLSX)

S2 Table Two-hundred-sixty-four metabolites detected across all groups. (XLSX)

S3 Table Fifty-two altered metabolites in the rats with DOX-CM. (XLSX)

S4 Table One-hundred-seven altered metabolites via SMI treatment. (XLSX)

## References

[1] Chatterjee K, Zhang J, Honbo N, Karliner JS (2010) Doxorubicin cardiomyopathy. Cardiology 115: 155–162. 10.1159/000265166 20016174 PMC2848530

[2] Octavia Y, Tocchetti CG, Gabrielson KL, Janssens S, Crijns HJ, Moens AL (2012) Doxorubicin-induced cardiomyopathy: from molecular mechanisms to therapeutic strategies. J Mol Cell Cardiol 52: 1213–1225. 10.1016/j.yjmcc.2012.03.006 22465037

[3] Sterba M, Popelova O, Vavrova A, Jirkovsky E, Kovarikova P, Gersl V, et al (2013) Oxidative stress, redox signaling, and metal chelation in anthracycline cardiotoxicity and pharmacological cardioprotection. Antioxid Redox Signal 18: 899–929. 10.1089/ars.2012.4795 22794198 PMC3557437

[4] Jang YM, Kendaiah S, Drew B, Phillips T, Selman C, Julian D, et al (2004) Doxorubicin treatment in vivo activates caspase-12 mediated cardiac apoptosis in both male and female rats. FEBS Letters 577: 483–490. 15556633 10.1016/j.febslet.2004.10.053

[5] Nicholson JK, Lindon JC (2008) Systems biology: metabonomics. Nature 455: 1054–1056. 10.1038/4551054a 18948945

[6] Rochfort S (2005) Metabolomics reviewed: a new "omics" platform technology for systems biology and implications for natural products research. J Nat Prod 68: 1813–1820. 16378385 10.1021/np050255w

[7] Putri SP, Nakayama Y, Matsuda F, Uchikata T, Kobayashi S, Matsubara A, et al (2013) Current metabolomics: practical applications. J Biosci Bioeng 115: 579–589. 10.1016/j.jbiosc.2012.12.007 23369275

[8] Robertson DG, Watkins PB, Reily MD (2011) Metabolomics in toxicology: preclinical and clinical applications. Toxicol Sci 120 Suppl 1: S146–170. 10.1093/toxsci/kfq358 21127352

[9] Wei R (2011) Metabolomics and its practical value in pharmaceutical industry. Curr Drug Metab 12: 345–358. 21395528 10.2174/138920011795202947

[10] Vinayavekhin N, Homan EA, Saghatelian A (2010) Exploring disease through metabolomics. ACS Chem Biol 5: 91–103. 10.1021/cb900271r 20020774

[11] Tan G, Lou Z, Liao W, Zhu Z, Dong X, Zhang W, et al (2011) Potential biomarkers in mouse myocardium of doxorubicin-induced cardiomyopathy: a metabonomic method and its application. PLoS One 6: e27683 10.1371/journal.pone.0027683 22110719 PMC3218026

[12] Zhou Q, Qin WZ, Liu SB, Kwong JS, Zhou J, Zhang W (2014) Shengmai (a traditional Chinese herbal medicine) for heart failure. Cochrane Database Syst Rev 4: Cd005052 10.1002/14651858.CD005052.pub5 24733159

[13] Chen L, Liang WX, Lv ZP (2010) [Synthetic evaluation of the clinical effect of the Shengmai capsule for treatment of chronic congestive heart failure using analytic hierarchy process]. Nan Fang Yi Ke Da Xue Xue Bao 30: 2036–2040. 20855244

[14] Zhang YC, Chen RM, Zhao MH (2002) [Effect of shengmai injection on hemodynamics in patients with dilated cardiomyopathy]. Zhongguo Zhong Xi Yi Jie He Za Zhi 22: 277–279. 12584790

[15] Zhang YK, He WB, Zhang H, He HY, Xie J, Xu JP (2007) [The observation of prevention effect of Shengmai injection for acute adriamycin induced cardiotoxicity]. Journal of Emergency in Traditional Chinese Medicine 16: 1082–1083.

[16] Yang XL (2008) [Clinical observation on the prevention of doxorubicin-associated cardiotoxicity by Shengmai injection]. Guide of China Medicine 6: 203–205.

[17] Li LH (2006) [Clinical study of the effects of Shengmai injection on induced myocardial toxicity]. Modern Journal of Integrated Traditional Chinese and Western Medicine 15: 595–596.

[18] Yang XD, Shu WQ, Yang SB (2008) [Protective effect of Shengmai injection on adriamycin-induced myocardium injury in rats and its mechanism]. Central South Pharmacy 6: 162–165.

[19] Jin H, Sun LM (2006) [Protective effects of Shengmai injection on myocardium injury induced by adriamycin in rats]. LiShiZhen Medicine and Materia Medica Research 17: 329–330.

[20] Zhang AH, Sun H, Qiu S, Wang XJ (2013) Recent Highlights of Metabolomics in Chinese Medicine Syndrome Research. Evid Based Complement Alternat Med 2013: 402159 10.1155/2013/402159 24302964 PMC3834606

[21] Wang X, Sun H, Zhang A, Sun W, Wang P, Wang Z (2011) Potential role of metabolomics apporoaches in the area of traditional Chinese medicine: as pillars of the bridge between Chinese and Western medicine. J Pharm Biomed Anal 55: 859–868. 10.1016/j.jpba.2011.01.042 21353755

[22] Zhang A, Sun H, Wang Z, Sun W, Wang P, Wang X (2010) Metabolomics: towards understanding traditional Chinese medicine. Planta Med 76: 2026–2035. 10.1055/s-0030-1250542 21058239

[23] Siveski-Iliskovic N, Kaul N, Singal PK (1994) Probucol promotes endogenous antioxidants and provides protection against adriamycin-induced cardiomyopathy in rats. Circulation 89: 2829–2835. 8205698 10.1161/01.cir.89.6.2829

[24] Kennedy LH, Sutter CH, Leon Carrion S, Tran QT, Bodreddigari S, Kensicki E, et al (2013) 2,3,7,8-Tetrachlorodibenzo-p-dioxin-mediated production of reactive oxygen species is an essential step in the mechanism of action to accelerate human keratinocyte differentiation. Toxicol Sci 132: 235–249. 10.1093/toxsci/kfs325 23152189 PMC3576006

[25] Dehaven CD, Evans AM, Dai H, Lawton KA (2010) Organization of GC/MS and LC/MS metabolomics data into chemical libraries. J Cheminform 2: 9 10.1186/1758-2946-2-9 20955607 PMC2984397

[26] Evans AM, DeHaven CD, Barrett T, Mitchell M, Milgram E (2009) Integrated, nontargeted ultrahigh performance liquid chromatography/electrospray ionization tandem mass spectrometry platform for the identification and relative quantification of the small-molecule complement of biological systems. Anal Chem 81: 6656–6667. 10.1021/ac901536h 19624122

[27] Thomas S, Bonchev D (2010) A survey of current software for network analysis in molecular biology. Hum Genomics 4: 353–360. 20650822 10.1186/1479-7364-4-5-353 PMC3500165

[28] van Bilsen M, Smeets PJ, Gilde AJ, van der Vusse GJ (2004) Metabolic remodelling of the failing heart: the cardiac burn-out syndrome? Cardiovasc Res 61: 218–226. 14736538 10.1016/j.cardiores.2003.11.014

[29] Ingwall JS (2009) Energy metabolism in heart failure and remodelling. Cardiovasc Res 81: 412–419. 10.1093/cvr/cvn301 18987051 PMC2639129

[30] Carvalho RA, Sousa RP, Cadete VJ, Lopaschuk GD, Palmeira CM, Bjork JA, et al (2010) Metabolic remodeling associated with subchronic doxorubicin cardiomyopathy. Toxicology 270: 92–98. 10.1016/j.tox.2010.01.019 20132857

[31] Katare R, Oikawa A, Cesselli D, Beltrami AP, Avolio E, Muthukrishnan D, et al (2013) Boosting the pentose phosphate pathway restores cardiac progenitor cell availability in diabetes. Cardiovasc Res 97: 55–65. 10.1093/cvr/cvs291 22997160 PMC3619276

[32] Kato T, Niizuma S, Inuzuka Y, Kawashima T, Okuda J, Tamaki Y, et al (2010) Analysis of Metabolic Remodeling in Compensated Left Ventricular Hypertrophy and Heart Failure. Circulation: Heart Failure 3: 420–430. 20176713 10.1161/CIRCHEARTFAILURE.109.888479

[33] Bordoni A, Biagi P, Hrelia S (1999) The impairment of essential fatty acid metabolism as a key factor in doxorubicin-induced damage in cultured rat cardiomyocytes. Biochimica et Biophysica Acta (BBA)—Molecular and Cell Biology of Lipids 1440: 100–106. 10477829 10.1016/s1388-1981(99)00113-4

[34] Subashini R, Ragavendran B, Gnanapragasam A, Kumar Yogeeta S, Devaki T (2007) Biochemical study on the protective potential of Nardostachys jatamansi extract on lipid profile and lipid metabolizing enzymes in doxorubicin intoxicated rats. Pharmazie 62: 382–387. 17557749

[35] Hong YM, Kim HS, Yoon HR (2002) Serum lipid and fatty acid profiles in adriamycin-treated rats after administration of L-carnitine. Pediatr Res 51: 249–255. 11809922 10.1203/00006450-200202000-00020

[36] Yao HT, Chang YW, Chen CT, Chiang MT, Chang L, Yeh TK (2008) Shengmai San reduces hepatic lipids and lipid peroxidation in rats fed on a high-cholesterol diet. J Ethnopharmacol 116: 49–57. 18162350 10.1016/j.jep.2007.10.043

[37] McNulty PH, Jacob R, Deckelbaum LI, Young LH (2000) Effect of hyperinsulinemia on myocardial amino acid uptake in patients with coronary artery disease. Metabolism 49: 1365–1369. 11079831 10.1053/meta.2000.9510

[38] Šimůnek T, Štěrba M, Popelová O, Adamcová M, Hrdina R, Geršl V (2009) Anthracycline-induced cardiotoxicity: Overview of studies examining the roles of oxidative stress and free cellular iron. Pharmacological Reports 61: 154–171. 19307704 10.1016/s1734-1140(09)70018-0

[39] Yin X, Wu H, Chen Y, Kang YJ (1998) Induction of antioxidants by adriamycin in mouse heart. Biochemical pharmacology 56: 87–93. 9698092 10.1016/s0006-2952(98)00099-9

[40] Jones SP, Greer JJ, Kakkar AK, Ware PD, Turnage RH, Hicks M, et al (2004) Endothelial nitric oxide synthase overexpression attenuates myocardial reperfusion injury. Am J Physiol Heart Circ Physiol 286: H276–282. 12969888 10.1152/ajpheart.00129.2003

[41] Calvert JW, Gundewar S, Jha S, Greer JJ, Bestermann WH, Tian R, et al (2008) Acute metformin therapy confers cardioprotection against myocardial infarction via AMPK-eNOS-mediated signaling. Diabetes 57: 696–705. 18083782 10.2337/db07-1098

[42] Lovric-Bencic M, Sikiric P, Hanzevacki JS, Seiwerth S, Rogic D, Kusec V, et al (2004) Doxorubicine-congestive heart failure-increased big endothelin-1 plasma concentration: reversal by amlodipine, losartan, and gastric pentadecapeptide BPC157 in rat and mouse. J Pharmacol Sci 95: 19–26. 15153646 10.1254/jphs.95.19

[43] Grimsrud PA, Xie H, Griffin TJ, Bernlohr DA (2008) Oxidative stress and covalent modification of protein with bioactive aldehydes. J Biol Chem 283: 21837–21841. 10.1074/jbc.R700019200 18445586 PMC2494933

[44] Mack CP, Hultquist DE, Shlafer M (1995) Myocardial flavin reductase and riboflavin: a potential role in decreasing reoxygenation injury. Biochemical and biophysical research communications 212: 35–40. 7612015 10.1006/bbrc.1995.1932

[45] Cox MJ, Sood HS, Hunt MJ, Chandler D, Henegar JR, Aru GM, et al (2002) Apoptosis in the left ventricle of chronic volume overload causes endocardial endothelial dysfunction in rats. Am J Physiol Heart Circ Physiol 282: H1197–1205. 11893552 10.1152/ajpheart.00483.2001

[46] Laplante M, Sabatini DM (2012) mTOR signaling in growth control and disease. Cell 149: 274–293. 10.1016/j.cell.2012.03.017 22500797 PMC3331679

[47] Choi YH, Jin GY, Li LC, Yan GH (2013) Inhibition of protein kinase C delta attenuates allergic airway inflammation through suppression of PI3K/Akt/mTOR/HIF-1 alpha/VEGF pathway. PLoS One 8: e81773 10.1371/journal.pone.0081773 24312355 PMC3843701

[48] Surh YJ, Kundu JK, Na HK (2008) Nrf2 as a master redox switch in turning on the cellular signaling involved in the induction of cytoprotective genes by some chemopreventive phytochemicals. Planta Med 74: 1526–1539. 10.1055/s-0028-1088302 18937164

[49] Chartoumpekis D, Ziros PG, Psyrogiannis A, Kyriazopoulou V, Papavassiliou AG, Habeos IG (2010) Simvastatin lowers reactive oxygen species level by Nrf2 activation via PI3K/Akt pathway. Biochem Biophys Res Commun 396: 463–466. 10.1016/j.bbrc.2010.04.117 20417615

[50] Ruiz S, Pergola PE, Zager RA, Vaziri ND (2013) Targeting the transcription factor Nrf2 to ameliorate oxidative stress and inflammation in chronic kidney disease. Kidney Int 83: 1029–1041. 10.1038/ki.2012.439 23325084 PMC3633725

[51] Ikeda Y, Aihara K, Akaike M, Sato T, Ishikawa K, Ise T, et al (2010) Androgen receptor counteracts Doxorubicin-induced cardiotoxicity in male mice. Mol Endocrinol 24: 1338–1348. 10.1210/me.2009-0402 20501642 PMC5417461

[52] Xiang P, Deng HY, Li K, Huang GY, Chen Y, Tu L, et al (2009) Dexrazoxane protects against doxorubicin-induced cardiomyopathy: upregulation of Akt and Erk phosphorylation in a rat model. Cancer Chemother Pharmacol 63: 343–349. 10.1007/s00280-008-0744-4 18379782

[53] Firdous AP, Kuttan R (2012) Chemo protective activity of carotenoid meso-zeaxanthin against doxorubicin-induced cardio toxicity. J Exp Ther Oncol 10: 101–106. 23350349

[54] Gnanapragasam A, Ebenezar KK, Sathish V, Govindaraju P, Devaki T (2004) Protective effect of Centella asiatica on antioxidant tissue defense system against adriamycin induced cardiomyopathy in rats. Life Sci 76: 585–597. 15556170 10.1016/j.lfs.2004.09.009

[55] Gnanapragasam A, Yogeeta S, Subhashini R, Ebenezar KK, Sathish V, Devaki T (2007) Adriamycin induced myocardial failure in rats: protective role of Centella asiatica. Mol Cell Biochem 294: 55–63. 16786185 10.1007/s11010-006-9245-0

[56] Shirwany NA, Zou MH (2010) AMPK in cardiovascular health and disease. Acta Pharmacol Sin 31: 1075–1084. 10.1038/aps.2010.139 20711221 PMC3078651

[57] Cardaci S, Filomeni G, Ciriolo MR (2012) Redox implications of AMPK-mediated signal transduction beyond energetic clues. J Cell Sci 125: 2115–2125. 10.1242/jcs.095216 22619229

[58] Cheng PW, Ho WY, Su YT, Lu PJ, Chen BZ, Cheng WH, et al (2014) Resveratrol decreases fructose-induced oxidative stress, mediated by NADPH oxidase via an AMPK-dependent mechanism. Br J Pharmacol 171: 2739–2750. 10.1111/bph.12648 24547812 PMC4243851

[59] Colombo SL, Moncada S (2009) AMPKalpha1 regulates the antioxidant status of vascular endothelial cells. Biochem J 421: 163–169. 10.1042/BJ20090613 19442239

[60] Wang S, Song P, Zou MH (2012) Inhibition of AMP-activated protein kinase alpha (AMPKalpha) by doxorubicin accentuates genotoxic stress and cell death in mouse embryonic fibroblasts and cardiomyocytes: role of p53 and SIRT1. J Biol Chem 287: 8001–8012. 10.1074/jbc.M111.315812 22267730 PMC3318690

[61] Ge M, Fang YY, Liu GP, Guan SD (2014) Effect of Shengmai injection on diaphragmatic contractility in doxorubicin-treated rats. Chin J Integr Med 20: 43–48. 10.1007/s11655-012-1096-9 22903440

